# Oil-in-Water Emulsions Probed Using Fluorescence Multivariate-Curve-Resolution Spectroscopy

**DOI:** 10.1021/acs.langmuir.4c01018

**Published:** 2024-06-11

**Authors:** Gülsüm Gündoğdu, Ezgi Yılmaz Topuzlu, Ferhat Mutlu, Umay E. Ertekin, Halil I. Okur

**Affiliations:** † Department of Chemistry, Bilkent University, 06800 Ankara, Turkey; ‡ National Nanotechnology Research Center (UNAM), Bilkent University, 06800 Ankara, Turkey; § Department of Energy Science and Technology, Faculty of Science, Turkish-German University, Istanbul 34820, Turkey

## Abstract

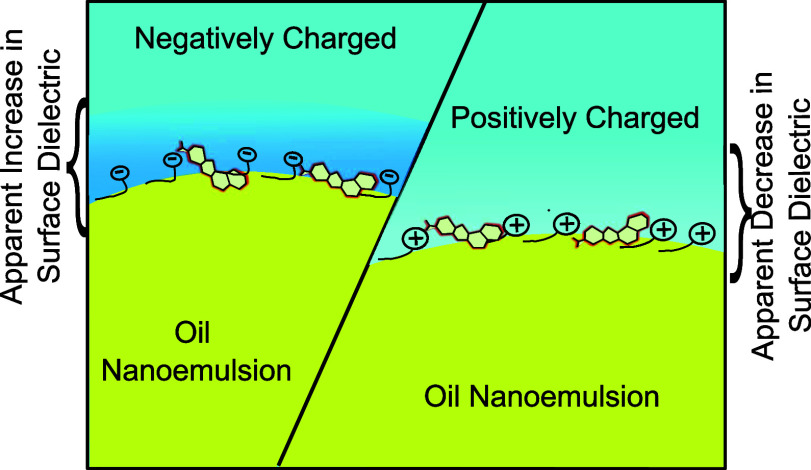

Hydrophobic surfaces in contact with aqueous media are omnipresent in nature. A plethora of key biological and physiological processes occur at the interface of immiscible fluids. Besides its fundamental importance, probing such interfaces is rather challenging, especially when one medium is bathed in the other. Herein, we demonstrate a fluorescence-based method that probes the oil–water interface and interfacial processes through surface dielectric perturbations. The fluorescence response of Nile Red is measured in hexadecane in water nanoemulsions. Three major spectral components appear: two from the bulk liquid media (hexadecane and water) and a distinct band at around 640 nm due to the interfacial component. Such spectra are deconvoluted using the multivariate-curve-resolution algorithm, and interface-correlated fluorescence spectra are attained. The influence of anionic sodium dodecylbenzenesulfonate (SDBS) and cationic cetyltrimethylammonium bromide (CTAB) surfactants on the oil–water interface is elucidated with concentration-dependent measurements. A charge-dependent spectral shift is observed. The interface correlated band at 641 nm for bare hexadecane nanoemulsions red shifts in the presence of anionic surfactants, indicating an apparent dielectric increase. In contrast, the same band gradually blue shifts with increasing cationic surfactant concentration, indicating an apparent interface dielectric decrease. Such a method can be utilized to probe alterations at interfaces beyond the oil/water interface.

## Introduction

**Figure 1:**
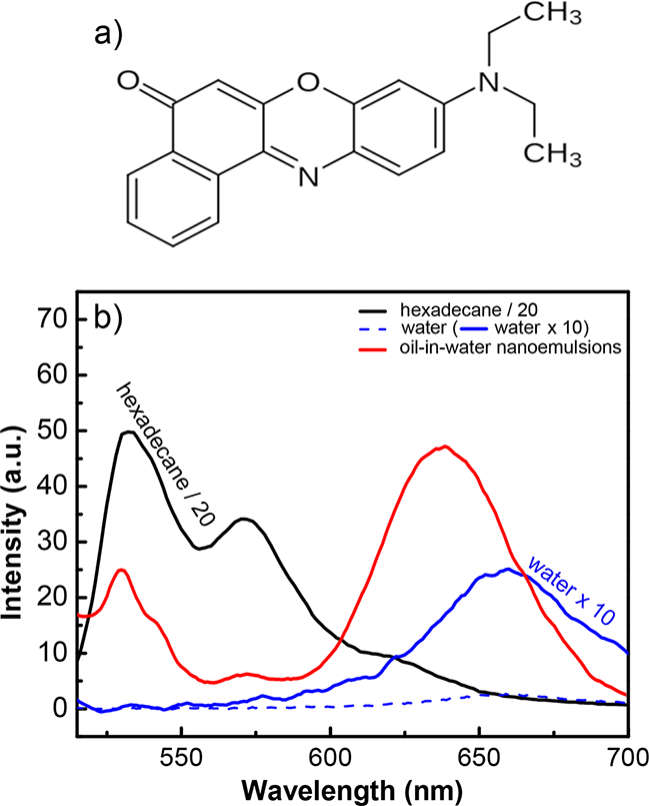
Fluorescence emission spectra of Nile Red in different environments. (a) Molecular structure of Nile Red dye. (b) Fluorescence emission spectra of the Nile Red molecule in pure hexadecane (black), water (dashed blue), and oil-in-water emulsion (red). Note that the blue solid spectrum displays 10× emission spectra of water. Excitation wavelength is 490 nm.

Hydrophobic media in contact with an aqueous solution are essential for life and host numerous heterogeneous reactions. Oil/water interfaces, lipid monolayers, and bilayers as well as emulsions are excellent examples of such medium.^1−3^ Due to their importance, in the last few decades, the molecular composition, structure, and dynamics of air/water and hydrophobic/water interfaces have been widely investigated as a model system via various experimental techniques^4−11^ and modeled by simulations.^12,13^ Namely, several spectroscopic techniques which include fluorescence spectroscopy were employed including UV–visible,^14^ IR and sum-frequency scattering,^15^ and fluorescence combined total internal reflectance (TIR)^16^ or external reflectance (ER),^17,18^ vibrational sum frequency (VSF),^19−23^ second harmonic generation (SHG),^24,25^ photothermal and laser spectroscopic methods.^26^ In particular the dielectric behavior of oil/water interfaces was investigated by dielectric spectroscopy,^27^ time-domain spectroscopy and resonance cavity methods,^28^ attenuated total reflection spectroscopy (ATR),^29^ and UV–vis absorption.^30^ Although many aspects of oil–water interfaces have been investigated, the interfacial dielectric properties remain mostly elusive. There are only a few reports on the interfacial polarity between two immiscible fluids.^31−33^ For example, the polarization at the oil/water interfaces was also investigated by means of time-resolved total-internal-reflection (TIR) fluorescence spectroscopy.^34^ Nile Red (Figure 1a), a fluorescence dye, is a sensitive probe of the change in the environment polarity. As reported before, it remains highly fluorescent within high concentrations of the oil nanodroplets.^35^

Herein, we introduced and characterized a new fluorescence-based methodology called Fluorescence Multivariate-Curve-Resolution (FMCR) Spectroscopy to probe the buried interfaces of soft material surfaces, i.e., oil-in-water nanoemulsion via interfacial dielectric perturbations. Nile Red (Figure 1a), a solvatochromic dye molecule, is employed to probe the dielectric perturbations of the oil/water interface of nanoscopic oil droplets in water. Three major spectral features are observed in the fluorescence spectrum for hexadecane and aqueous media, along with a new spectral feature for the oil/water interface. A concentration-dependent sizable spectral shift is observed in its fluorescence emission band due to the dielectric perturbation of the adsorption of negatively charged sodium dodecylbenzenesulfonate (SDBS) or positively charged cationic cetyltrimethylammonium bromide (CTAB) surfactants at the oil/water interface. By deconvolution of the spectral components, interfacial components can be investigated. The spectroscopic method provides information on interfacial dielectric perturbation at the oil/water interfaces; by applying a self-modeling Multivariate-Curve-Resolution (SMCR) algorithm, the sole contribution of the oil/water interface can be extracted. These fluorescence spectra of the Nile Red molecule are shown to shift based on the polarity change of the environment where the dye molecule is solvated.

## Results and Discussion

In the first set of experiments, the fluorescence spectra of Nile Red in different environments are measured. Figure 1b shows the fluorescence spectra of various solvents where a minute amount of Nile Red dye (0.12 μM) is introduced. Namely, pure hexadecane (black) and an aqueous solution (dashed blue) are utilized as sole solvents. It gives rise to spectral features with two distinct emission peaks at 532 and 571 nm in hexadecane, a low dielectric medium. These two bands were assigned to planar, and bent molecular conformations, respectively.^36^ In an aqueous solution, the fluorescence spectra are different, with lower fluorescence quantum yielding a new broad spectral feature rising at 660 nm (dashed blue, and 10× blue curve). Surprisingly, the fluorescence spectrum of a hexadecane oil-in-water nanoemulsion is quite different. A new band at 640 nm arises from the Nile Red molecules absorbed at the oil–water interfaces when the nanoemulsions are present. Nile Red is known to respond to dielectric changes via a shift in the fluorescence emission band. For instance, it has been routinely used to visualize lipid bodies and lipid vesicles in fluorescence imaging of cell samples.^37^ Nile Red in hexadecane nanoemulsions in water stabilized with surfactants give rise to three distinct fluorescence bands including two bands at 532 and 571 nm, as well as a new emission band at 640 nm. The former two peaks can be assigned to Nile Red molecules partitioned to the hydrophobic-low dielectric phase. Note that the spectral features have the same frequency with bulk hexadecane medium. The latter spectral feature, however, should not be due to either of the bulk phases (hexadecane and water). This band should be due to the absorbed dye molecules at the oil/water interface. To verify that the new band at 640 nm is due to the hexadecane in the water interface of the oil-nanoemulsion, a set of control experiments is performed. First, the new band at nearly 640 nm is only observed when the oil/water interface, i.e., nanoemulsions, is present. More importantly, the interface band responds to known influences on the oil/water interface of nanoemulsions. For instance, the surface band shows a spectral shift in the presence of surface-active tetraphenylborate ions, whereas no apparent shift occurs in the presence of NaCl, a nonsurface-active salt (see SI-2 and Figure S3 for details). As such, the fluorescence band at 640 nm in Figure 1b can be assigned to an emission peak reporting on the interfacial dielectric environment. More specifically, the peak position can be employed to probe the dielectric properties of the oil/water interface. Nile Red as a neutral molecule would minimally influence any charge involved in interfacial processes and thus can be employed as a new method to probe interfacial processes through changes in the local dielectric. Next, the utilization of the band for the surface adsorption of surfactant molecules will be demonstrated.

The surface dielectric sensitive interfacial fluorescence band (∼640 nm) makes two main contributions. The response of Nile Red is due to the dielectric of the interfacial region and the interference of the fluorescence bands of the low dielectric media contributions. The latter contribution can significantly alter the position and width of the interfacial band. That can be eliminated and pure interface-correlated spectrum can be achieved via the self-modeling multivariate-curve-resolution (SMCR) algorithm. This approach deconvolutes the interface-correlated fluorescence spectrum. MCR algorithm has been successfully employed to separate different contributions of the data in other measurements such as chromatography, and NMR spectroscopy.^39,40^ More recently, it was also utilized to attain the solvent-correlated vibrational spectrum of the O–H stretching region for both infrared and Raman spectroscopy.^41−51^ For the nanoemulsion samples, the fluorescence spectra of a series of nanodroplet samples with different Nile Red concentrations are measured, and the same interfacial peak is observed (see Figure S4). An SMCR algorithm is applied as described in the SI-1. Briefly, an SMCR algorithm diminishes the maximum amount of reference spectral features from the measured sample spectrum. The resulting spectra can be employed to report on the surface dielectric and the dielectric changes with any interfacial processes.

**Figure 2:**
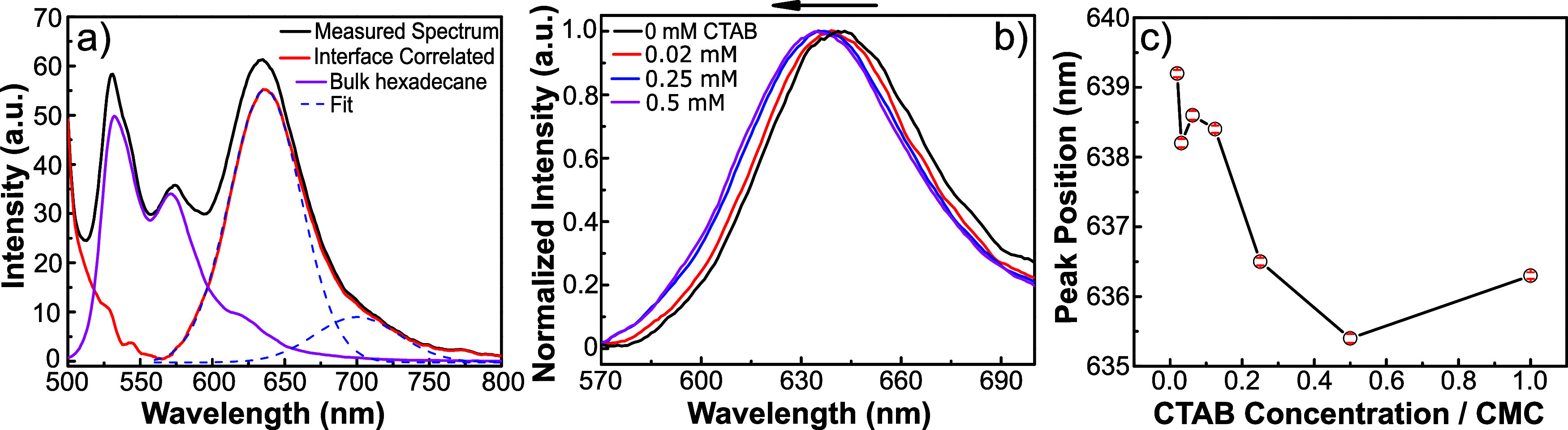
(a) Fluorescence spectrum of a nanoemulsion sample with 0.25 mM CTAB (black line), Nile Red in bulk hexadecane spectrum (pink line), and interface correlated spectrum extracted by using SMCR algorithm (red line). The interface correlated spectrum fit using two Gaussian bands (blue dashed line). (b) Change in the interfacial correlated peak as the CTAB concentration increases showing the hypochromic shift. (c) Peak position with increasing normalized concentrations up to 0.5 CTAB concentration/CMC, where the CMC is taken as 1.0 mM.^38^

Figure 2a shows the measured spectrum from the nanoemulsion sample with 0.25 mM CTAB (black line), Nile Red in bulk hexadecane spectrum (pink line), and interface correlated spectrum extracted by using the SMCR (red line) algorithm. It can be clearly seen that the SMCR process removes the additional contributions from the interfacial band in the spectral region of 550 nm−800 nm. The band seen below 550 nm is assigned to light scattering from the nanoemulsion solution. The deconvoluted interface correlated spectrum (red line) has an apparent 1.3 nm red shift in the peak position compared to the measured spectrum. Note that there are still two contributions to the interface-correlated spectrum and these can be separated by fitting to two Gaussian bands. These contributions are from the interface, and bulk water, in which the pure water band is a broad band with a low emission coefficient. Thus, its contribution to interfacial peak position and peak width is only minimal, if there are any. Figure 2b shows how the interface-correlated band can be employed to probe buried interfaces. Nanoemulsions with cationic cetyltrimethylammonium bromide (CTAB) surfactants at different concentrations are measured including a surfactant-free sample (Figure 2b). Note that, the surfactant-free nanoemulsions are significantly less stable compared to the other samples, and thus all the measurements related to surfactant-free nanoemulsions were performed within the first few hours after preparation. The interfacial peak position alters as the CTAB concentration increases. As bulk CTAB concentration increases, the surface composition at the oil/water interface also changes. It was shown that more surfactants adsorb to the oil/water interface of nanoemulsions as a function of surfactant concentration.^52−55^ It results in a blue shift for interface correlated peak position. To demonstrate the trend in the spectral shift, the peak position with respect to surfactant concentration is plotted and a sharp decrease up to 0.5× of CTAB critical micelle concentration (CMC), followed up by a plato can be observed (Figure 2c). Surfactant concentrations above CMC create surfactant micelles and can cause additional surfactant bands (see SI-3 and Figure S4c). Next, the surface adsorption of different surfactants and their influence on surface polarity are tested. Hence, cetylpyridinium bromide (CBH), another positively charged surfactant, is also tested with nanoemulsion solutions, and a similar spectral shift is observed (see SI-4 and Figure S5). These data indicate that the interfacial band position reports on surface charge-induced interfacial polarity changes at the oil/water interface of nanoemulsions.

**Figure 3:**
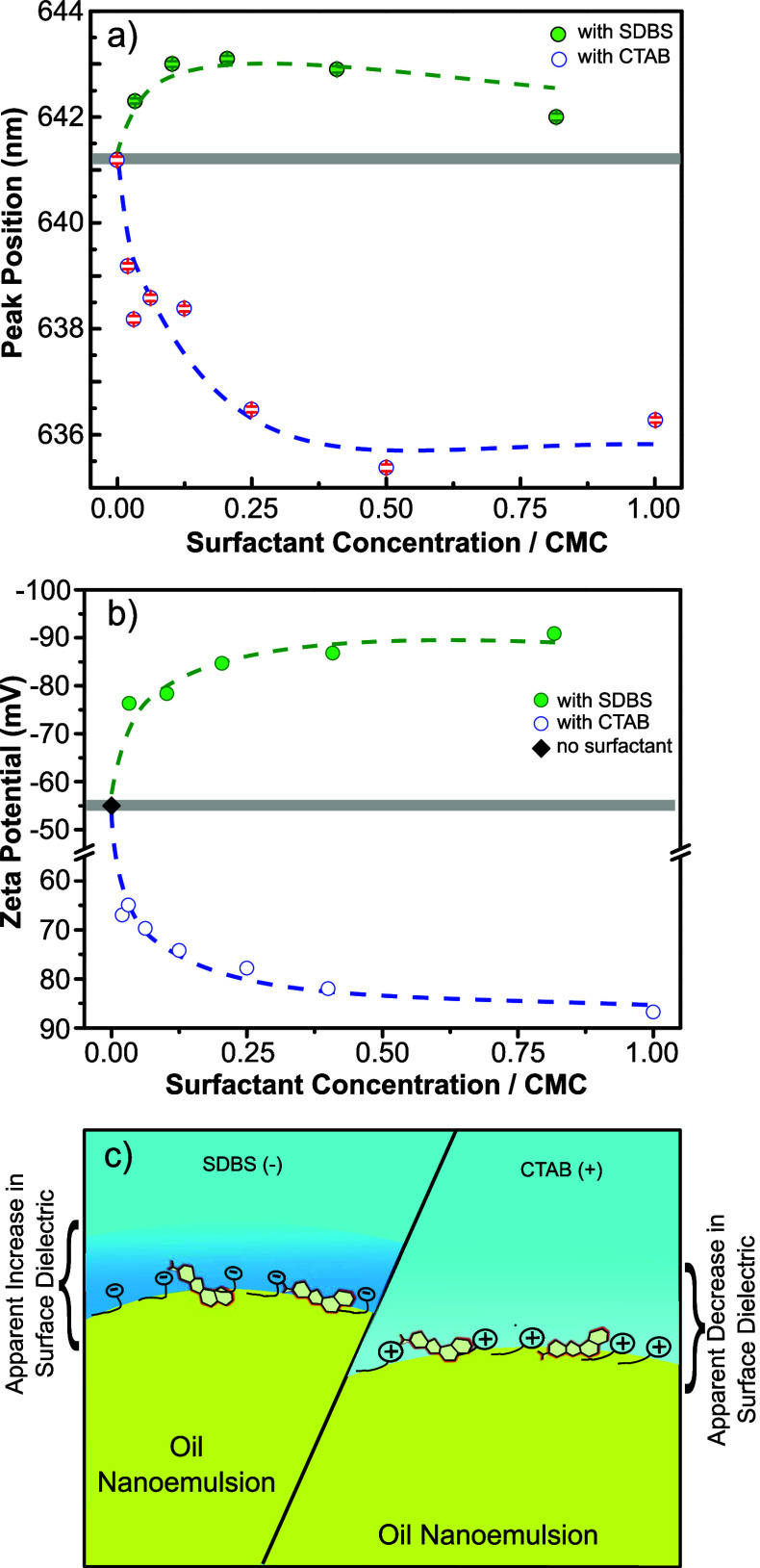
(a) Interface correlated fluorescence peak position change for hexadecane nanoemulsions as a function of surfactant concentration for CTAB and SDBS. (b) Zeta potential values of the nanoemulsion samples with CTAB and SDBS with increasing the concentration at pH 7. (c) Schematic picture of the effect of charged surfactants on interfacial dielectric both for SDBS and CTAB. For simplicity, the counterions are not shown in the illustration.

In the next set of experiments, nanoemulsions with the negatively charged surfactant, i.e., sodium dodecylbenzenesulfonate (SDBS), with varying concentrations are measured. Figure 3a shows the peak positions of nanoemulsions in the presence of CTAB and SDBS surfactants as a function of normalized surfactant concentration. In contrast to nanoemulsions with CTAB, a minor concentration-dependent red shift (∼2 nm) in the interface-correlated band is observed. The data suggests that the interfacial dielectric change with charged surfactant affects in opposite directions. The cationic headgroups decrease the interfacial dielectric, whereas the sulfate-based anionic headgroups increase the interfacial dielectric. In order to demonstrate the link between the surface charge and interfacial dielectric, the zeta potential (ζ) of the nanoemulsions is performed. The bare nanoemulsions with no surfactants have a ζ-potential of −55 mV. This recurring value is in accordance with proposed mechanisms^8,10,56^ and agreement with the literature.^57,58^ As a function of negatively charged SDBS concentration, ζ-potential value gradually becomes more negative and reaches −91 mV. On the contrary, nanoemulsions with positively charged surfactants give rise to a significant increase and reach a zeta potential value of +87 mV. Although zeta potential is not exactly probing the surface potential, the value is mostly correlated with the surface potential.^59^ Thus, the surface potential moves in opposite directions with these surfactants. With negatively charged surfactants it decreases ∼36 mV more, whereas with cationic surfactants it increases ∼142 mV. The ratio is in line with the peak shift of the interfacial band of Nile Red (Figure 3a). The charged surfactants alter the apparent interfacial dielectric of the oil nanoemulsions. The cationic ones decrease the apparent surface dielectric, whereas the anionic ones increase it. Yet, the interfacial picture is case-dependent and could be more complex than just the sign of the charge. Surfactant headgroup chemistry, charge density, and hydration properties should play a role in the overall surface dielectric alteration. Nevertheless, the surfactants between CTAB and CBH for cationic, and SDBS and SDS for anionic ones yield quite similar trends (see SI-4, Figure S5). A schematic description of the proposed interface and related apparent surface dielectric change is shown in Figure 3c. The demonstrated method shown in this study can be adapted to explore other soft-material interfaces, such as polymersomes, liposomes, and lipid bodies.

## Conclusions

In this study, we show a fluorescence-based method probing the oil–water interface and interfacial processes through surface dielectric perturbations. By measuring the fluorescence response of Nile Red in pure liquid hexadecane, in aqueous media, as well as oil-in-water nanoemulsions, a clear interface correlated spectral band is demonstrated. In hexadecane nanoemulsion samples, three major spectral components appear; two from the bulk liquid media (hexadecane, water) and a distinct interface-related band at ∼640 nm. By using the self-modeling multivariate curve-resolution algorithm, interface-correlated fluorescence spectra are attained. The influence of anionic sodium dodecylbenzenesulfonate (SDBS) and cationic cetyltrimethylammonium bromide (CTAB) surfactants on the oil–water interface is explored. A charge-dependent spectral shift is observed. Surprisingly, the interface correlated band red shifts in the presence of anionic surfactants, indicating an apparent dielectric increase, and a gradual blue shift with increasing cationic surfactant concentration, indicating an apparent interface dielectric decrease. These results are confirmed for other charged surfactants (sodium dodecyl sulfate and cetylpyridinium bromide hydrate) as well. The proposed method in this study can be utilized to elucidate other soft-material interfaces, such as polymersomes, liposomes, lipid bodies, and others.

## Materials and Methods

### Chemicals

Hexadecane (Sigma-Aldrich, 99.9%, analytical grade), sodium dodecyl sulfate (SDS) (Sigma-Aldrich, technical grade), sodium dodecylbenzenesulfonate (SDBS) (Sigma-Aldrich, technical grade), cetyltrimethylammonium bromide (CTAB) (Sigma-Aldrich, ≥96.0% (AT)), cetylpyridinium bromide hydrate (CBH) (Sigma-Aldrich, 98%), and methanol (Sigma-Aldrich, ACS reagent, ≥99.8%) along with Nile Red (Sigma-Aldrich) were all purchased from Sigma-Aldrich and used as received. Millipore deionized water (resistivity: 18 MΩ cm^–1^) was used in all of the experiments.

### Nanoemulsions

Oil nanoemulsions which consist of bare oil nanodroplets were prepared in aqueous solution with a known protocol of was prepared with anionic surfactants sodium dodecyl sulfonate (SDS) and sodium dodecylbenzenesulfonate (SDBS) and with cationic surfactants cetyltrimethylammonium bromide (CTAB) and cetylpyridinium bromide hydrate (CBH) at different concentrations below their critical micelle concentration.^60−62^ 2% hexadecane is added to an aqueous solution that was mixed for 5 min with a hand-held homogenizer (TH, OMNI International) and subsequently placed in an ultrasonic bath (35 kHz, 400 W, Bandelin) for the same duration.^38,63,64^ The average size, size distribution (PDI), and zeta-potential of the nanodroplets were measured with dynamic light scattering (DLS, Malvern ZS nanosizer). The nanodroplets were consistently found to have a mean diameter in the range of 200–300 nm with a polydispersity index (PDI) of less than 0.25. See SI-5 and Figure S6 for details. The nanoemulsion size could not be altered in a controlled manner for kinetically stabilized oil-in-water emulsions; therefore, size-dependent fluorescence measurements were not performed.

### Measurements

The nanoemulsion samples were diluted to 1/60 ratio with corresponding surfactant solution (water for bare nanoemulsion samples), and Nile Red solution was added to reach a final concentration of 0.12 μM. The samples were transferred to disposable UV cuvettes. The fluorescence measurements were carried out via a Varian Cary Eclipse Fluorescence Spectrometer. Measurements were performed with 490 nm excitation wavelength and 500–800 nm spectral window for the emission.

## Supporting Information Available

More details about the multivariate curve resolution analysis (PDF)
