# Supplementary material for: Oil-in-Water Emulsions Probed Using Fluorescence Multivariate-Curve-Resolution Spectroscopy
Source: Langmuir. 2024 Jun 11;40(25):13116–21. doi: 10.1021/acs.langmuir.4c01018 (PMC11494642; doi:10.1021/acs.langmuir.4c01018)
Supplement: Supplementary file 1 — la4c01018_si_001.pdf [file la4c01018_si_001.pdf]

SUPPORTING INFORMATION

Oil in Water Emulsions Probed using Fluorescence Multivariate-Curve-Resolution Spectroscopy

Gülsüm Gündoğdu[1,2,3], Ezgi Yılmaz Topuzlu[1,2], Ferhat Mutlu[1], Umay E. Ertekin[1], Halil I. Okur*[1,2]

[1] Department of Chemistry, Bilkent University, 06800 Ankara, Turkey

[2] National Nanotechnology Research Center (UNAM), Bilkent University, 06800 Ankara, Turkey

[3] Department of Energy Science and Technology, Faculty of Science, Turkish-German University, Istanbul 34820, Turkey

SI – 1 Self modeling Multivariate Curve Resolution Algorithm

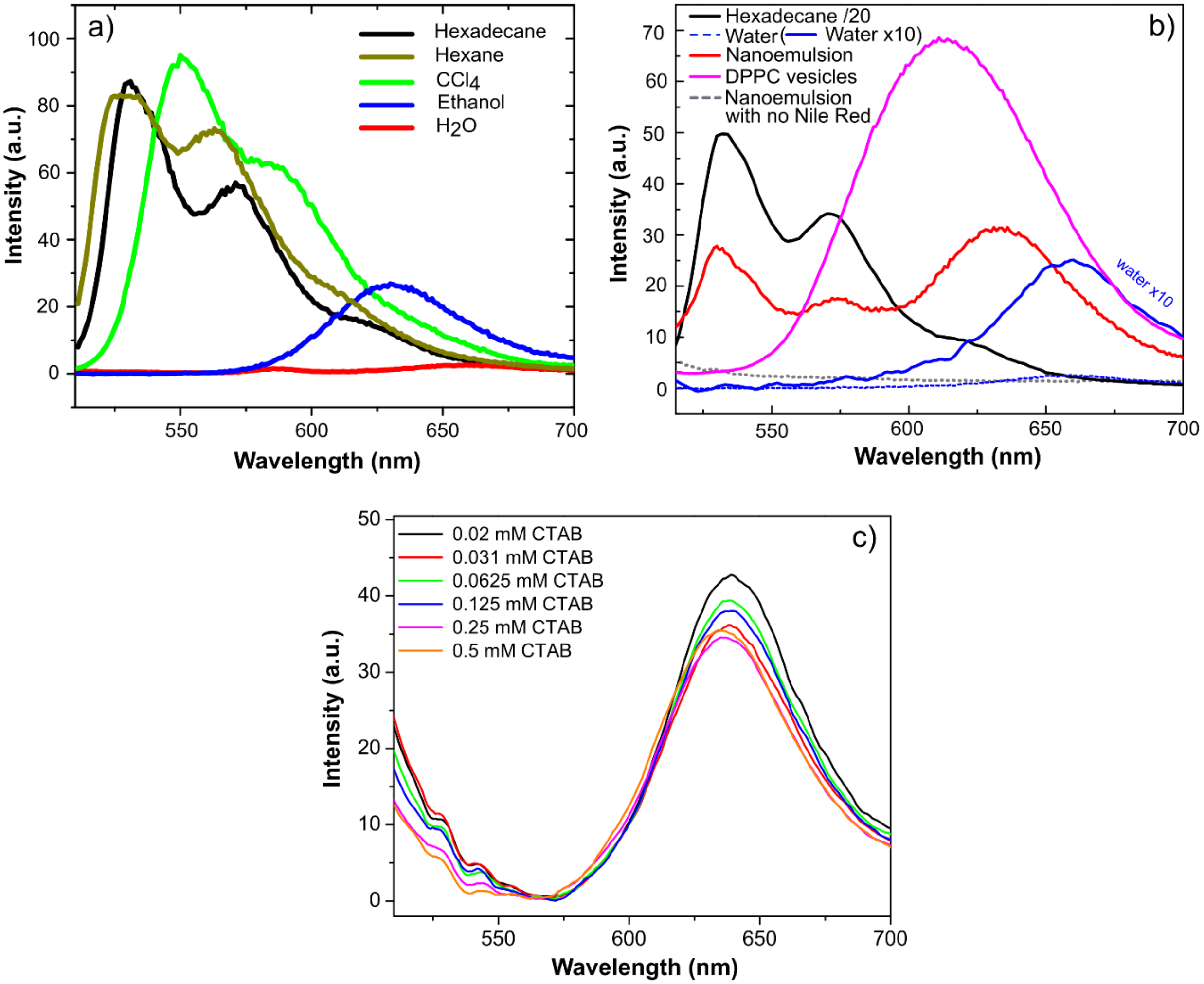

Figure S1. a) Fluorescence response of solvatochromic dye, Nile Red, in the solvents of hexadecane, hexane, carbon tetrachloride, ethanol, and water. b) Fluorescence response of Nile Red in bulk hexadecane (black, divided by 20), in bulk water, in water/oil interface (red) and in DPPC vesicles (magenta). The Fluorescence response of nanoemulsion with no Nile-Red is also shown (gray dashed line). c) The FMCR spectrum of interfacial fluorescence band as a function of CTAB concentration.

The fluorescence response of solvatochromic dye molecules, i.e. Nile Red, in various solvents is recorded. Figure S1a shows the spectra of Nile Red in hexadecane, hexane, carbon tetrachloride (CCl_4), ethanol and water. The response of Nile Red to the dielectric of the medium can be seen clearly in the spectra. At a low dielectric environment, two spectral features for linear and bend structure of Nile Red are observed. When the solvent dielectric of the medium increases, it gives rise to a single band which gradually red shifts. The spectral response of Nile Red is also tested

against more complex media, such as oil-in-water nanoemulsions, and dipalmitoyl phosphatidylchloride (DPPC) vesicles, see Figure S1b. In the spectra of the multi-component and complex nanoemulsion system, there are mainly three different contributions; bulk hexadecane, bulk aqueous solution, and the interface of the nanoemulsions. Fluorescence response of different concentration CTAB in Nile Red is demonstrated in Figure S1c. No monotonic intensity trend has been seen. In order to obtain the interface-correlated spectrum for each measurement, that is associated with the oil/water interface, the data analysis is carried out with self-modeling multivariate curve resolution (SMCR) method.

Self-modeling curve resolution (SMCR) method is a well-suited tool for the deconvolution spectroscopic data and is based on the bilinear model.[1]

$$X = CS^T + E \qquad (1)$$

The goal of SMCR is to monitor the extraction of concentration profiles matrix C of the relevant pure component spectral profiles matrix S from the measured data matrix X (Eqn 1). The part of the spectra unexplained by the pure components forms the residual matrix E. The decomposition of X is achieved by iteratively minimizing the residuals of E under suitable boundary conditions. The matrices C and S are initially estimated. Using the least squares method or weighted least squares, C and S are then calculated iteratively, to reach the bilinear model that gives the best fit. The calculation step is carried out until reaching a predetermined convergence.[1]

The first and the key step in SMCR-based analysis of data is to determine the number of pure variable components, which have a contribution to the multicomponent signal. There is a large number of SMCR-based methods that can be applied. For this study, we used Self-Modeling Multivariate Curve Resolution by Alternating Least-Squares (SMCR-ALS). ALS as an optimization method that enables SMCR to incorporate any data-specific constraint. With the opportunity to use prior knowledge as the boundary conditions, makes SMCR-ALS easy-to-apply for multi-component systems. The SMCR method plays an essential role in deconvoluting each component in a system.[1–3] This method requires two spectra as inputs; one of them is the reference spectrum (bulk hexadecane), which will be subtracted, and the other one is the spectrum of the sample itself. The algorithm enables to see the fluorescence response originated from the interface after the contribution of bulk hexadecane was subtracted.

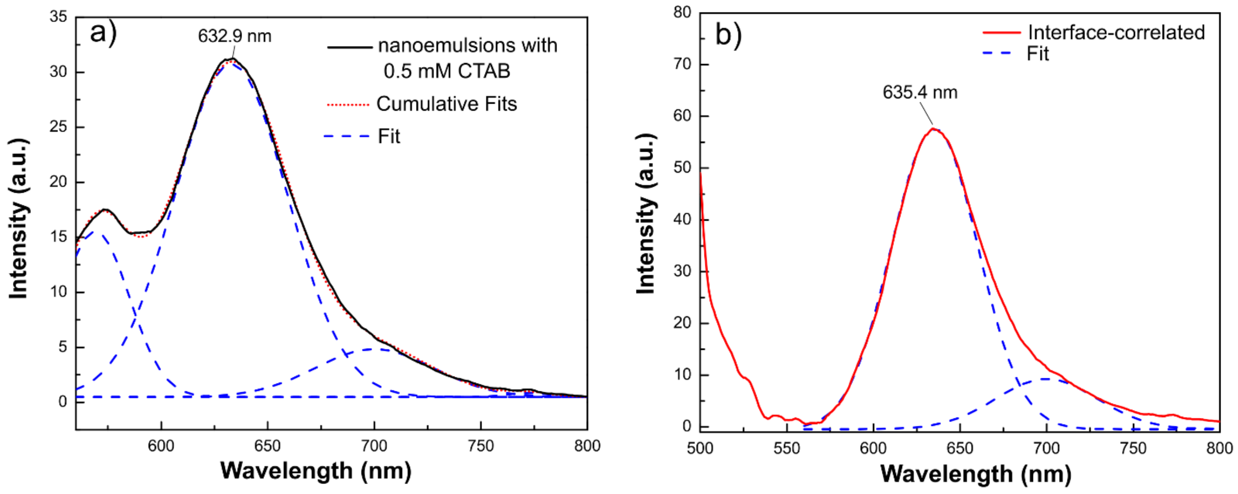

Figure S2. a) Fitted raw spectrum of sample with 0.5 mM CTAB before SMCR with main peak position at 632.9 nm. b) Fitted spectrum of sample with 0.5 mM CTAB after SMCR with main peak position at 635.4 nm.

In order to emphasize the necessity to apply SMCR process, the surface-correlated band is fitted to a gaussian function before the SMCR process (Figure S2a), and after (Figure S2b) for hexadecane nanoemulsion sample stabilized with 0.5 mM CTAB surfactants. Without SMCR, the actual peak position cannot be found without interference with other bands. The sample with 0.5 mM CTAB is measured and gaussian fitting is applied to find the peak position for the main peak from the interface by having an additional peak at constant 700 nm. Before the SMCR process, the peak position is obtained at 632.9 nm. However, the spectra after the SMCR process, interface-correlated data are obtained by removing the contributions from bulk hexadecane, and this time the peak position is observed at 635.4 nm. An overall, 2.5 nm spectral shift occurs. Considering the degree of shifts seen in the main paper, achieving the actual peak positions via SMCR process is critical.

SI – 2 Nile Red to probe interfaces

Nile Red dye molecule at the interface shows an emission band that can be correlated to the interface of the oil-in-water nanoemulsions. In order to elucidate whether the band actually interface related, a set of experiments are performed. At first, instead of soft nanoemulsions, colloidal beads with approximately 300 nm in diameter are utilized as the buried interface in aqueous environment. As seen in Figure S3a both polystyrene, and polystyrene sulfate beads show no surface emission bands due to the surface adsorption of the Nile Red dye molecule at the interface. The small emission band seen for both samples at 650 nm, which should be related

to the bulk aqueous solution. This data suggests that Nile Red can probe the interface of soft-material surfaces, and also cannot probe the interface when the surface is made of solid material like polystyrene. In a second set of experiments, nanoemulsions containing Nile Red are tested in the presence of different salts. Surface inactive (sodium chloride) and surface active (sodium tetraphenylborate) salts were utilized. Figure S3b & S3c show the fluorescence spectra of these nanoemulsion samples. The interface-correlated band shifts with the presence of surface-active (BPh_4^-) ions, and in the presence of surface inactive salt (NaCl) the band remains at its original position. Results indicate that the interface assigned band can probe the changes at the interface. When the surface of oil-in-water nanoemulsions are modified with the surface-active ions, the interface-correlated band gives rise to a substantial shift, whereas no apparent shift was present for the NaCl salt.

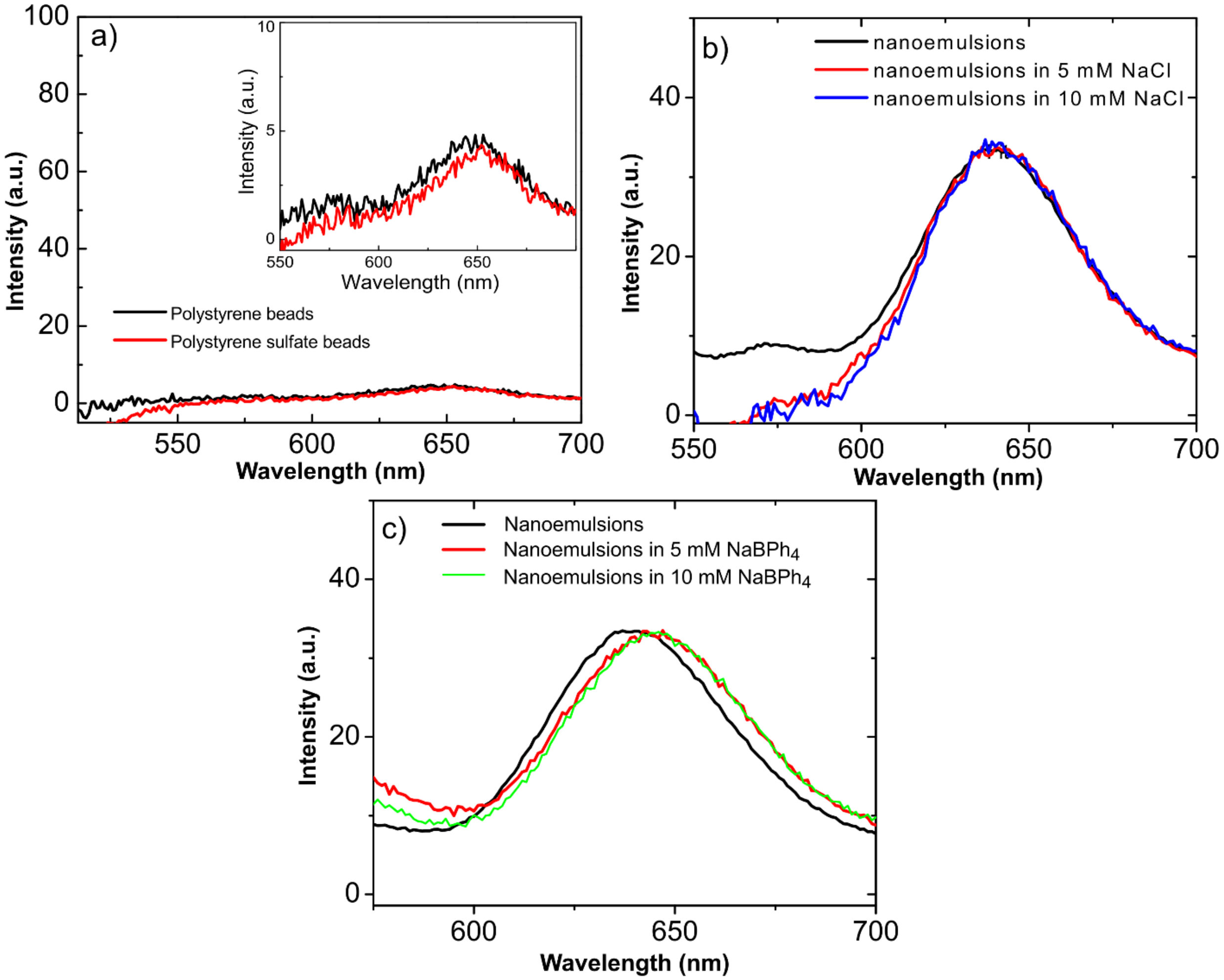

Figure S3. a) Fluorescence spectra of Nile Red with polystyrene, and polystyrene sulfate beads. The inset shows the same spectra with higher resolution on the intensity axis. b) Fluorescence spectra Nile Red for samples of hexadecane nanoemulsions in aqueous solutions of different

sodium chloride (NaCl) concentrations. c) Fluorescence spectra Nile Red for samples of hexadecane nanoemulsions in aqueous solutions of different sodium tetraphenylborate ($NaBPh_4$) concentrations.

SI – 3 Nile Red concentration and excitation frequency dependence

The amount of added Nile Red does not influence the wavelength but the intensity of the interface-correlated emission band. Figure S4a shows the fluorescence spectra of the hexadecane nanoemulsions with different final Nile Red concentrations. The effect of excitation frequency is also tested. In each data, 490 nm is employed as excitation frequency. As can be seen from Figure S4b, although the florescence intensity alters with excitation wavelength, peak position remains unaltered.

In order to further elucidate the underlying reason for the appearance of ~ 640 nm interfacial fluorescence band, a set of samples identical to nanoemulsions, except the hexadecane oil was removed, were measured (See Figure S4c). Around the critical micelle concentration (CMC) of the surfactant, where the micelles and surfactant aggregates form, some fluorescence intensity can be seen from the Nile Red at 635 nm. Yet, at lower surfactant concentrations, almost no fluorescence intensity can be observed. Note that the fluorescence intensity of surfactant solution is similar to that of surfactant free water sample. Moreover, adding hexadecane oil and forming the nanoemulsions forms quite high hydrophobic surface area that compete with the free surfactant aggregates by their surface adsorption affinity. Thus, the presence of nanoemulsions decreases the effective free surfactant concentrations. As such, at the surfactant concentration range focused in this study (below CMC), only surfactant aggregate related fluorescence band remains negligible. In Figure S4d, the florescence spectra of Nile Red in pure hexadecane, and hexadecane with 0.25/CMC CTAB without the aqueous phase were measured. No apparent change has been seen for hexadecane and hexadecane with 0.25/CMC CTAB, especially above 600 nm.

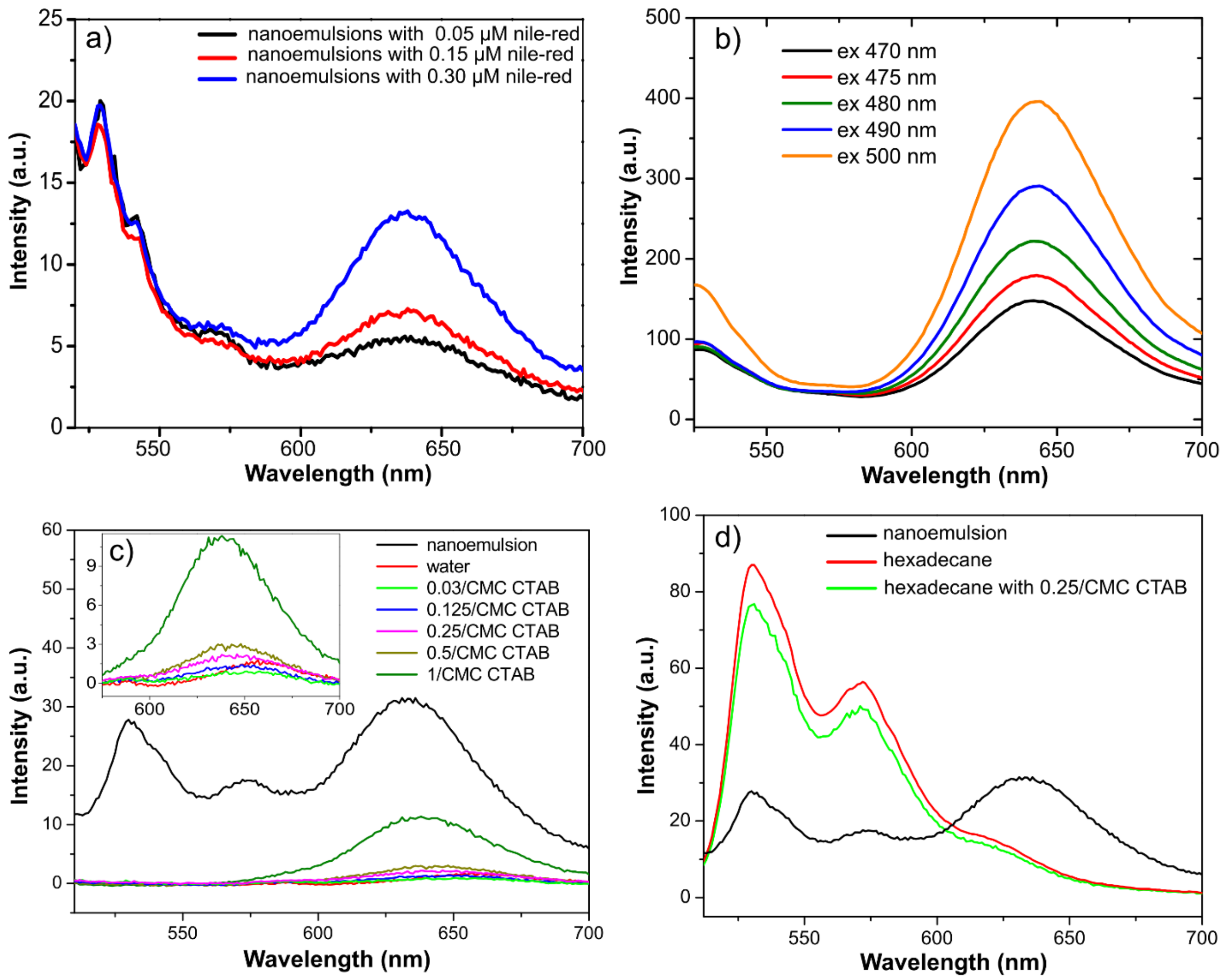

Figure S4. a) The fluorescence spectra of Nile Red nanoemulsions at different Nile Red concentrations from 0.05 to 0.30 μM concentration. b) The fluorescence spectra of Nile Red in nanoemulsions sample at different excitation frequencies. c) The florescence spectra of Nile Red as a function of surfactant in the absence of hexadecane oil. Nanoemulsion, and water spectra are added for refence to eye. The inset shows the spectral region at 580 – 700 nm. d) The florescence spectra of Nile Red in pure hexadecane, and hexadecane with 0.25/CMC CTAB without aqueous phase.

SI – 4 Measuring Other Surfactants

The surfactant dependent fluorescence measurements are performed for additional surfactants; cetylpyridinium bromide hydrate (CBH) and sodium dodecyl sulfate (SDS). The interface related band peak position as function of surfactant concentration can be seen Figure S5a & 5b. Namely, the trends observed for cationic and anionic surfactants remain identical as described in the main text.

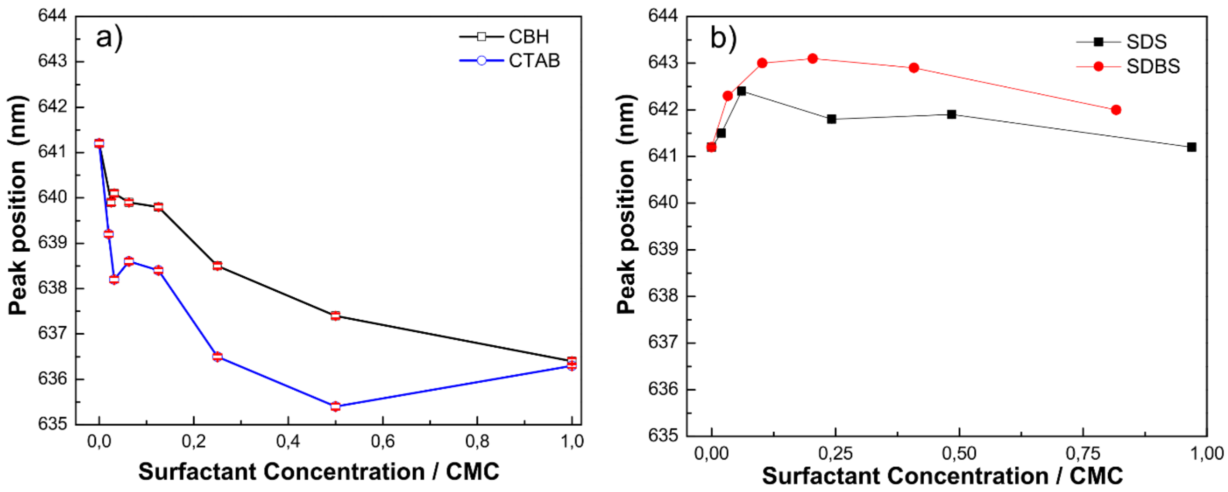

Figure S5. a) The graph for interface-correlated emission band peak position as a function of normalized surfactant concentration for positively charged surfactants; CTAB and cetylpyridinium bromide hydrate (CBH). b) The graph for interface-correlated emission band peak position as a function of normalized surfactant concentration for negatively charged surfactants; SDBS and sodium dodecyl sulfate (SDS).

SI – 5 Droplet stability, size distribution and zeta-potential measurements

The size distribution and zeta-potential of the nanodroplets were measured by dynamic light scattering (DLS, Zetasizer Nano-ZS) with He-Ne laser 633 nm, as a light source, and the measurements were performed at room temperature. The nanoemulsion sample placed in a disposable cuvette and the DLS measurements were usually repeated at least three times to ensure reproducibility of results and the average values were reported. The nanoemulsion samples with different concentrations were consistently found to have a mean diameter in the range of 200-300 nm with a polydispersity index (PDI) of less than 0.3. Figure S6 shows a representative size distribution measurement that is performed for a nanoemulsion sample containing 0.0025 mM CTAB surfactant. The average diameter is 204.1 nm with a size distribution; PDI value of 0.326 and the corresponding zeta potential is 67.8 mV. The general size

distribution as well as the PDI values are in line with this reported measurement.

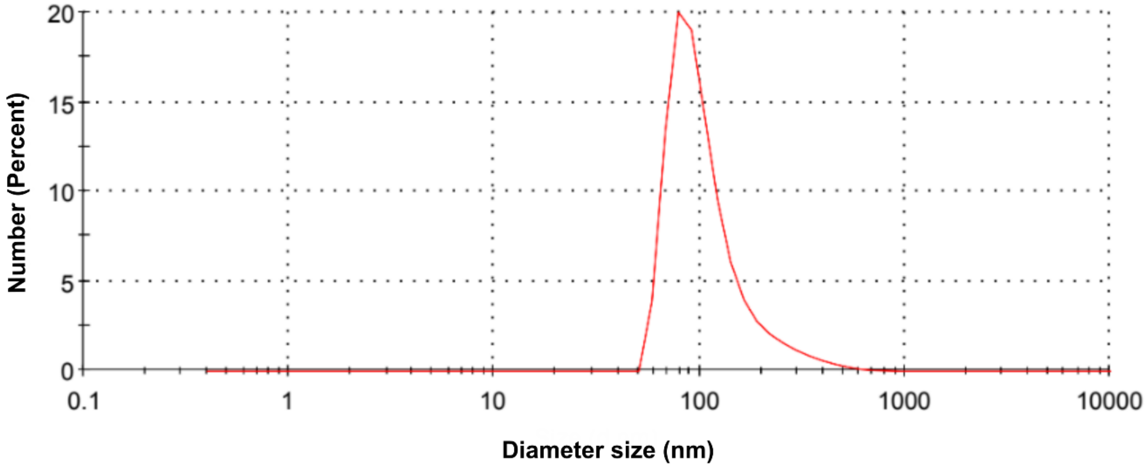

Figure S6. Nanoemulsion stability as a function of size distribution by number of a sample containing 0.0025 mM CTAB concentration. This size distribution curve is representative for nanoemulsions samples containing various surfactants.

References

(1) Jiang, J. H.; Ozaki, Y. Self-Modeli Curve Resolution (SMCR): Principles, Techniques, and Applications. *Applied Spectroscopy Reviews* **2002**, *37* (3), 321–345.

(2) Lawton, W. H.; Sylvestre, E. A. Self Modeling Curve Resolution. *Technometrics* **1971**, *13* (3), 617–633.

(3) Tefera, D. T.; Agrawal, A.; Yañez Jaramillo, L. M.; De Klerk, A.; Prasad, V. Self-Modeling Multivariate Curve Resolution Model for Online Monitoring of Bitumen Conversion Using Infrared Spectroscopy. *Industrial and Engineering Chemistry Research* **2017**, *56* (38), 10756–10769.
